# The Impact of Saffron on Symptoms of Withdrawal Syndrome in Patients Undergoing Maintenance Treatment for Opioid Addiction in Sabzevar Parish in 2017

**DOI:** 10.1155/2017/1079132

**Published:** 2017-11-21

**Authors:** Mohammad Nemat Shahi, Atefeh Asadi, Elham Behnam Talab, Mahbobeh Nemat Shahi

**Affiliations:** ^1^Sabzevar University of Medical Sciences, Abaresh, Iran; ^2^Health Department, Sabzevar University of Medical Sciences, Abaresh, Iran

## Abstract

**Background and Objective:**

Drug dependence is one of the serious problems around the world. Saffron is one of those beneficial medicinal plants which is embedded with a similar mechanism to methadone (e.g., inhibition of serotonin reuptake). Thus, it can be helpful in reducing the withdrawal symptoms. The aim of this study was to reduce the daily dose of methadone usage and reduce its side effects using saffron in the form of medicinal supplements.

**Methodology:**

This study was categorized as a clinical trial. Accordingly, 44 clients of addiction treatment centers in Sabzevar parish were randomly selected to participate in this study in 2016–2017. While the experimental group was treated with methadone syrup and self-made saffron capsules, the control group received methadone syrup and placebo capsules.

**Results:**

The results showed that the use of saffron and methadone alleviated the symptoms of withdrawal syndrome (*p<0*.001).

**Conclusion:**

Having reviewed the research participants, it was indicated that the introduction of saffron alleviated the symptoms of withdrawal syndrome in patients undergoing maintenance treatment for opioid addiction. Thus, it seems rational to make use of saffron in combination with methadone in order to alleviate the symptoms of withdrawal syndrome.

## 1. Introduction

Nowadays, the main mechanisms and brain-driven factors that are associated with opioid dependence are known to a large extent. It seems that the dopamine mesocorticolimbic pathway is one of the most important pathways associated with drug dependence that play an important and vital role in psychological dependence to opioids [1]. This pathway starts from the ventral tegmental area, which is considered the A10 dopamine neurons, and ends in nucleus accumbens, frontal cortex, and other areas involved in memory, such as the hippocampus and amygdala. However, other anatomical pathways and neurotransmitters have been identified in the brain which may be at least attributed to a portion of the opioid-induced psychological dependence. Since opioid drugs stimulate dopamine release in the mesocorticolimbic pathways, it is argued that these drugs can also induce long-term memory [3].

The withdrawal syndrome is a syndrome which occurs when blood or tissue concentrations of a substance drop in an individual who has used large amounts of the substance for a long time. Although the symptoms of each substance are unique, there are some similar symptoms (e.g., substance carving) [4]. The importance of withdrawal syndrome may be inferred through more probable relapse; the patient's distress; immediate health risks (cardiovascular instability); mental disorders associated with withdrawal (such as depression induced by opioids) [5]; physical symptoms including tremor, muscle cramps, bone and muscle pain, chill, sweating, rhinitis, flu-like symptoms, diarrhea, yawning, sneezing, tachycardia, etc.; psychological symptoms, including dysphoria, depression, anxiety, or panic attacks; dizziness; agitation; as well as rare but serious symptoms, including cardiac arrhythmia, stroke, seizure, and suicide [6, 7].

The latter can be diagnosed through interview and clinical examination. However, the urine test may be used to confirm the diagnosis [8].

The onset of withdrawal symptoms for short-acting opioids, such as morphine and heroin, lasts from 1 to 7 days, which starts within 8 to 12 hours after the last consumption and reaches the maximum level up to 48 hours. Given the long-acting opioids, such as methadone, it is observed that the onset of withdrawal symptoms lasts from 7 to 10 days and reaches the maximum level up to 3 to 8 days, and these symptoms continue up to few weeks. It should be noted that its nonspecific binding to tissues generates a great source of methadone in the body. This source prevents from rapid drug drop-off and, thus, it is rational to prescribe it once a day [9, 10].

The treatment of withdrawal or deprivation syndrome consists of providing supportive therapy, including treatment of fluid and electrolyte disorders, and paying due attention to the associated disorders, such as anxiety or depression. However, the main treatment is based on such long-acting opioids as methadone. If the patient feels pain, nonsteroidal anti-inflammatory drugs (NSAIDs) are preferred, but if there is a medical prohibition (e.g., stomach ulcer), one can increase the methadone dose. Besides, some ancillary medications such as clonidine, trazodone, and ondansetron are used for symptomatic treatment [11].

The inhibition of serotonin reuptake is one of the mechanisms of methadone through which the addict's general and mental conditions are improved. Similarly, saffron is characterized with the same mechanism and, additionally, it is embedded with anti-inflammatory and analgesic effects. Since the forenamed effects have been confirmed throughout the previous studies, it may be argued that saffron is highly recommended to be used as an ancillary medication to reduce the dose of methadone. In this paper, it was attempted to investigate and compare the effect of saffron and placebo on patients undergoing methadone treatment [12, 13].

Since herbal medicines are characterized with fewer side effects, they have been considered as ideal alternatives for chemical drugs in the past few decades, and they are prescribed in a rising trend [14]. Saffron is one of the plants that have been used as a medicinal plant since ancient times in different parts of the world [15]. In Iran, this plant has been used as an antispasmodic, tranquilizer, digestion facilitator, antiflatulence, sudatory, mucous, pain-relieving, pain and sexual-arousal medication [14].

Saffron is full of fatty substances, minerals, and mucilage. Other compounds include a colorless essence consisting of terpenes and cineol. It should be noted that the smell of saffron is related to these materials. Picrocrocin is a bitter heteroside which easily dissolves in water and alcohol and brings up a glocoside whose hydrolysis generates glucose and picrocrocin. Crocin is a carotenoid that constitutes the main component of saffron [16]. It is supposed that the antidepressant effect of saffron stems from crocin and safranal located in the stigma. Crocin functions as a water-soluble compound and safranal functions as a fat-soluble compound. Accordingly, they are embedded with two different mechanisms. It is postulated that crocin affects the dopaminergic system and norepinephrine reuptake inhibition, and safranal affects the serotonergic system [14]. A new study suggests that crocin (major alkaloid of saffron) boosts the level of dopamine in the striatum and prevents the destruction of dopamine neurons [17].

## 2. Materials and Methods

This study was categorized as a clinical trial. Accordingly, clients of addiction treatment centers in Sabzevar parish were randomly selected to participate in this study in 2016–2017. Given the clients of addiction treatment centers whose files were available in the forenamed centers in Sabzevar parish (it should be noted that all participants were located in the stability phase, and they received a fixed dose of methadone for several months and did not represent any mental and physical symptoms), it was attempted to randomly select the eligible individuals using computer software. Next, participants were divided into two groups. Similarly, treatment was randomly assigned to groups in that secretary of each center, who was unaware of the content of cards named as A and B, delivered the cards to the selected participants. When the participants visited the center, the cards were checked by a physician at each center and, subsequently, the desired treatment was done based on the type of the concerned card (holders of Card A and Card B received a self-made saffron capsule and a placebo capsule, resp.). Before the start of drug intervention, a specific checklist was filled for each patient. The forenamed checklists contained some items on the absence of any withdrawal symptoms (lack of any signs of physical and psychological withdrawal symptoms and independence to increased dose of methadone was considered the research inclusion criterion). While the experimental group was treated with methadone syrup and self-made saffron capsules (each saffron capsule contained 30 mg of saffron powder), the control group received methadone syrup and placebo capsules (which were similar to saffron capsules in terms of color and size). The saffron and placebo capsules were prescribed by the researcher once a week for 8 weeks in the clinic (exactly at the delivery time of weekly dose of methadone), and the patients were forced to consume them at the same location.

Given the decrease in methadone dose during the study period (5 mg per week) and its replacement with self-made saffron capsules (30 mg of saffron powder or placebo capsule per week), it was decided to provide telephone lines for the patients around the clock. Besides, they were instructed that, in case of advent of any (physical or mental) withdrawal symptoms, they could call their corresponding physician so that they would stop taking the capsules and receive increased dose of methadone. Furthermore, the checklists were weekly and in-personally filled in line with patients' symptoms, and in case of advent of withdrawal symptoms, the patients were checked in terms of compliance with the treatment regimen and recommendations. If it was diagnosed that the patients did not have the ability to continue the process of intervention, they would be excluded from the research. Otherwise, the patients would be checked again, and they would be located in their original group. Some distinct individuals, who were unaware of the type of treatment, undertook to control the patients' symptoms, collect the required data, and evaluate the results. Finally, the results were analyzed using SPSS Software (Version 20). It should be noted that none of the patients met the exclusion criteria.

## 3. Results

Regarding the two participating groups in this study, it was indicated that the mean age of participants was 39.55 years. Besides, the minimum age and maximum age were designated as 21 and 67 years, respectively.

The mean age of participants in the experimental and control groups was designated as 41.5 and 37.5 years, respectively.

Regarding the occupation of the participants, it was declared that 72.7% of participants in the experimental group were self-employed. However, 63.6% of participants in the control group were self-employed (Table 1).

**Figure 1:**
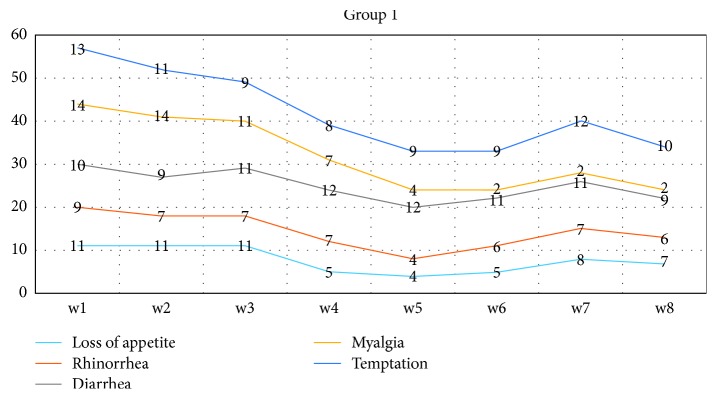
Experimental.

**Figure 2:**
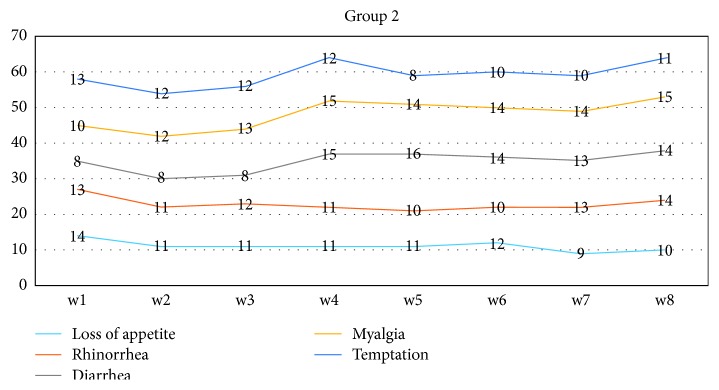
Control.

The results showed that the status of subjects in connection to withdrawal symptoms might be depicted in terms of Figures 1 and 2.

Given the experimental group, it was observed that the status of “loss of appetite” moved from 50% at the end of the first month to 31.8% at the end of the second month. Besides, it was found that loss of appetite was on a downward trend at the beginning of the fourth week. Regarding the control group, it was indicated that there was no change in the sign of loss of appetite, and it was almost constant (Figure 2).

Given the status of rhinorrhea in Group 1 (experimental), there was observed a lagging downward trend. This downward trend moved from 40% at baseline to 27% at the end of the second month, and the forenamed trend peaked at the beginning of the second month (18%). Regarding Group 2 (control), it was observed that the rising trend moved from 59% to 63% thereof.

Given the status of diarrhea in Group 1, it was indicated that the downward trend moved from 50% to 40% at the end of the second month. Regarding Group 2, it was observed that the rising trend moved from 36% at the beginning of the first week to 63% at the end of the eighth week.

There was a serious downward trend in terms of myalgia in Group 1. Actually, the aforementioned trend moved from 63% at baseline to 9% at the end of eighth week, and this serious trend took a quicker step at the start of the second month. Regarding the control group, it was observed that the changing and rising trend moved from 40% at the baseline to 68% at the end of the second month.

The results indicated that the status of temptation in Group 1 was faced with a downward trend and moved from 59% to 50% throughout the research participants. Then, a fairly stable trend was seen from the third to the sixth week. Conversely, there was not a systematic downward trend in the control group.

## 4. Discussion and Conclusion

Having conducted a two-month investigation on the research participants, it was revealed that addiction withdrawal symptoms in patients undergoing the saffron-based maintenance treatment were alleviated. The lifetime prevalence of drug abuse was about 20 percent [18]. Regarding this fact that drug abuse was considered as one of the most important problems in the realm of public health, the authorities responsible for developing and promoting health in the society were recommended to adopt solutions to combat this challenge and its associated problems. To do this end, this study aimed to investigate the impact of saffron on symptoms of withdrawal syndrome in patients undergoing maintenance treatment for opioid addiction. Then, 100 participants willingly participated in the study.

Their age range was between 21 and 67 years, and the majority of participants were young. Tavakoli et al. conducted a study on higher percentage of middle-aged participants. However, the reason for the difference in results stemmed from two different statistical populations. Given a study conducted in Hamedan, it was observed that the average age was about 30 years, which was consistent with the results of the current study. It seems that the addiction dilemma can encompass the majority of age groups and, thus, this latter issue deserves due attention. All participants in this study were male patients, which correspond with the results of a study conducted by Hojjati et al. It seems that addiction is more prevalent in men than women [12].

Methadone may be considered among effective drug treatment methods in connection to addiction treatment. Since methadone is effective in eliminating withdrawal symptoms of drug discontinuation for 24 hours or more, it may be argued that methadone is useful for the treatment of addiction [13].

Methadone is one of the most effective opioid analgesics that affect the central nervous system, and it generates such effects as analgesia, euphoria, and tranquility [18].

In this study, five major symptoms of withdrawal, diarrhea, rhinorrhea, myalgia, temptation, and loss of appetite, were investigated.

Regarding the research results and having compared the forenamed results with other similar studies, it was concluded that saffron-based treatment was effective in alleviating the symptoms of withdrawal syndrome in patients undergoing maintenance treatment for opioid addiction. Besides, it was indicated that similar previous studies were in line with the results of the current study.

## Figures and Tables

**Table 1:** 

Measurement unit (%)	Group 1 (experimental)	Group 2 (control)	Total number of participants
Jobholder	9.1	9.1	9
Self-employed	72.7	63.6	68
Unemployed	18.2	27.3	22.7
