# Corrigendum to “The Impact of Saffron on Symptoms of Withdrawal Syndrome in Patients Undergoing Maintenance Treatment for Opioid Addiction in Sabzevar Parish in 2017”

**DOI:** 10.1155/2020/9208647

**Published:** 2020-09-19

**Authors:** Mohammad Nematshahi, Atefeh Asadi, Elham Behnamtalab, Mahbobeh Nematshahi

**Affiliations:** ^1^Sabzevar University of Medical Sciences, Sabzevar, Iran; ^2^Health Department, Sabzevar University of Medical Sciences, Sabzevar, Iran

In the article titled “The Impact of Saffron on Symptoms of Withdrawal Syndrome in Patients Undergoing Maintenance Treatment for Opioid Addiction in Sabzevar Parish in 2017” [1], the affiliation should be changed from “Sabzevar University of Medical Sciences, Abaresh, Iran” to the correct affiliation “Sabzevar University of Medical Sciences, Sabzevar, Iran.”

The correct list of affiliations is shown above.
